# Hunt for Palytoxins in a Wide Variety of Marine Organisms Harvested in 2010 on the French Mediterranean Coast

**DOI:** 10.3390/md13085425

**Published:** 2015-08-21

**Authors:** Ronel Biré, Sophie Trotereau, Rodolphe Lemée, Davide Oregioni, Christine Delpont, Sophie Krys, Thierry Guérin

**Affiliations:** 1 University of Paris-Est, ANSES, Maisons-Alfort Laboratory for Food Safety, Department of Chemical Contaminants in Food, Pesticides and Marine Biotoxins Unit, 14 rue Pierre et Marie Curie, 94706 Maisons-Alfort Cedex, France; E-Mails: sophie.trotereau@anses.fr (S.T.); christine.delpont@anses.fr (C.D.); sophie.krys@anses.fr (S.K.); thierry.guerin@anses.fr (T.G.); 2 Observatoire Océanologique de Villefranche sur mer, Université Pierre et Marie Curie, Laboratoire d’Océanographie de Villefranche CNRS UMR 7093, 181 Chemin du Lazaret 06230 Villefranche-sur-mer, France; E-Mails: lemee@obs-vlfr.fr (R.L.); oregioni@geoazur.unice.fr (D.O.)

**Keywords:** *Ostreopsis*, palytoxins-group toxins, Mediterranean sea, marine organisms, haemolytic test, LC-MS/MS

## Abstract

During the summer of 2010, 31 species including fish, echinoderms, gastropods, crustaceans, cephalopods and sponges were sampled in the Bay of Villefranche on the French Mediterranean coast and screened for the presence of PLTX-group toxins using the haemolytic assay. Liquid chromatography tandem mass spectrometry (LC-MS/MS) was used for confirmatory purposes and to determine the toxin profile. The mean toxin concentration in the whole flesh of all sampled marine organisms, determined using the lower- (LB) and upper-bound (UB) approach was 4.3 and 5.1 µg·kg^−1^, respectively, with less than 1% of the results exceeding the European Food Safety Authority (EFSA) threshold of 30 µg·kg^−1^ and the highest values being reported for sea urchins (107.6 and 108.0 µg·kg^−1^). Toxins accumulated almost exclusively in the digestive tube of the tested species, with the exception of octopus, in which there were detectable toxin amounts in the remaining tissues (RT). The mean toxin concentration in the RT of the sampled organisms (fishes, echinoderms and cephalopods) was 0.7 and 1.7 µg·kg^−1^ (LB and UB, respectively), with a maximum value of 19.9 µg·kg^−1^ for octopus RT. The herbivorous and omnivorous organisms were the most contaminated species, indicating that diet influences the contamination process, and the LC-MS/MS revealed that ovatoxin-a was the only toxin detected.

## 1. Introduction

Palytoxin (PLTX) is a potent non-protein marine compound produced by corals of the genus *Palythoa* [1,2,3] and by dinoflagellates of the genus *Ostreopsis* (in which the compound is referred to as putative PLTX, p-PLTX) [4,5]. Several PLTX analogues have been identified so far, either from *Palythoa* (homoPLTX, bishomoPLTX, neoPLTX and deoxyPLTX [6], 42-hydroxy-PLTX [3], PLTX-b [7]) or from *Ostreopsis* (Ovatoxins (OVTX) a to h [5,8,9,10,11,12], OVTX-a AC, -b AC, -d AC and -e AC [13], ostreocins [14,15], and mascarenotoxins [7,16,17]).

Since the early 2000s, *Ostreopsis* blooms have been reported with increasing frequency worldwide, including in the Mediterranean Sea [18,19]. In September 2006, divers and swimmers around the Frioul Islands, off Marseilles, reported various symptoms related to the presence of *Ostreopsis* in the water [20]. Since then, a monitoring system has been set up by the competent authority along the French Mediterranean coast; when *Ostreopsis* abundances exceed 4000 cells/L in the water column, shellfish are analysed by liquid chromatography tandem mass spectrometry (LC-MS/MS) to quantify the presence of PLTX-group toxins [4]. Currently, there are no regulations for these toxins in the European Union or elsewhere. However, the European Food Safety Authority (EFSA), based on a risk assessment, recommends a threshold of 30 µg·kg^−1^ [21]. Only limited data on a few species (sea urchins, mussels and clams) were available at the time, including those provided from the French national surveillance network for phytoplankton and phycotoxins (REPHY) run by the French Research Institute for Exploitation of the Sea (IFREMER). There is therefore a need for additional occurrence data to enhance the risk assessment and to transpose it to a wider range of marine organisms.

Various studies have been conducted on the French Mediterranean coast to determine the contamination levels in p-PLTX and its analogues in various marine organisms, such as mussels, sea urchins, crustaceans, gastropods, cephalopods and different species of fishes [4,22,23]. These studies have shown that some of the sampled species, namely mussels, sea urchins and certain fishes can have toxin levels above the EFSA-recommended threshold, with the toxins being mainly located in the digestive tube and rarely in the remaining tissues (flesh) [4,23]. Such findings, if confirmed and extended to a wider range of organisms, could be used as a basis for risk management strategies (e.g., evisceration), to protect seafood consumers.

The aim of this work was to collect information on the contamination levels in p-PLTX and its analogues in a wide range of marine organisms harvested weekly in summer 2010, in the presence of an *Ostreopsis* cf. *ovata* bloom, and to study the toxin distribution. This was the first time that so many species were analysed for this purpose (31 species including fishes, crustaceans, bivalve molluscs, gastropods, echinoderms and cephalopods).

## 2. Results and Discussion

### 2.1. Evolution of the O. cf. ovata Abundances and Toxin Concentrations in Marine Organisms

**Figure 1:**
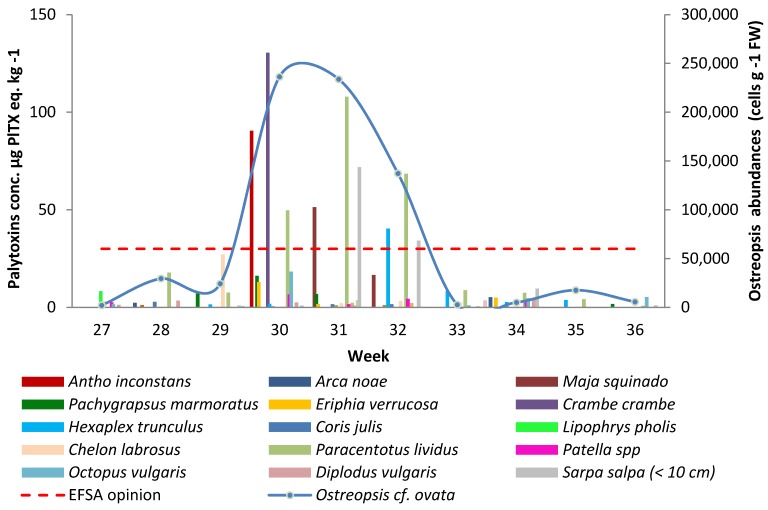
Palytoxin concentration determined by the haemolytic test in the whole flesh of the marine organisms harvested in Rochambeau from week 27 to week 36, and calculated using the lower bound (LB) approach. The dashed line represents the EFSA threshold of 30 µg PLTX + OST-D·kg^−1^. The blue curve represents the abundance of epiphytic *Ostreopsis* cells over the same period.

Rochambeau was selected as a sampling site given its history of *O.* cf. *ovata* presence in the water column and as an epiphyte on macroalgae. *O.* cf. *ovata* cells were monitored on site for 10 weeks in the summer of 2010. The abundance of the epiphytic cells began to rise from week 27, with 2105 cells·g^−1^ fresh weight (FW), reached a maximum in week 30 with 236,276 cells·g^−1^ FW and then decreased to 5672 cells·g^−1^ FW by week 36 (Figure 1). During the *Ostreopsis* bloom, cell counts were greater than 1 million cells·g^−1^ FW in some sampling points. The epiphytic abundances in the present study were, in average, more than four times higher than in the 2009 study [23] at the same location. However, monitoring in the previous study only started in Rochambeau in week 32 (early August 2009), and cell counts continued to decrease until the end of the sampling period, in week 36 (early September 2009). Independent data collected as part of the MediOs 2 research programme showed that *Ostreopsis* abundances in Rochambeau in 2009 ranged from *ca*. 193,000 to 794,000 cells·g^−1^ FW between weeks 29 and 31, with the highest counts being recorded in week 30 (Lemée, pers. Comm.). The monitoring of the sampling sites of Villefranche-sur-Mer (“Plage de la reserve” and “Plage des jeunes”) and Nice in 2009 showed that the maximum epiphytic cell abundances were recorded in week 31 [23]. This peak in abundance appears to occur regularly during the first fortnight of August, as the maximum values reported in Rochambeau in 2009, and in 2010 (present study) were observed in weeks 31 and 32. Brissard *et al.* [4] reported that the epiphytic cell abundances in the Bay of Villefranche-sur-Mer peaked at the end of July 2011 (week 30), further corroborating that mid-summer is the main blooming period in the north-west Mediterranean [18,24].

Several environmental parameters can affect the growth of *O.* cf. *ovata* [18,24,25]; water depth may play an important role because epiphytic abundance decreases with depth [4,26]. The sampling depth (0.5 m) used in the present study corresponded to that at which the highest abundance was observed in Brissard *et al.* [4].

The abundance pattern in the water column (expressed in cells·L^−1^) perfectly matched that of the epiphytic fraction, with a maximum concentration of 4750 cells·L^−1^ reached in week 31 (Figure S1), a phenomenon also observed in 2009 [23].

The marine organisms harvested in Rochambeau were exposed to high abundances of *Ostreopsis* cells between weeks 30 and 32. The recorded toxin levels showed a similar pattern: the haemolytic test gave the highest concentrations in the same time period for the red-encrusting sponge *Antho inconstans* (week 30; 90.5 µg PLTX eq.·kg^−1^ whole flesh [WF]), the orange-red encrusting sponge *Crambe crambe* (week 30; 130.5 µg PLTX eq.·kg^−1^), the spinous spider crab *Maja squinado* (week 31; 51.3 µg PLTX eq.·kg^−1^), the sea urchin *Paracentrotus lividus* (week 31; 107.6 µg PLTX eq.·kg^−1^), juvenile Salema porgy *Sarpa salpa* under 10 cm in size (week 31; 71.8 µg PLTX eq.·kg^−1^), and the banded dye-murex *Hexaplex trunculus* (week 32; 40.4 µg PLTX eq.·kg^−1^) (Figure 1).

The above-mentioned species were the only six species, of the 31 species sampled, that reached a maximum WF toxin concentration above 30 µg PLTX eq.·kg^−1^. The pattern of contamination observed in the marine organisms perfectly matched the evolution of the *Ostreopsis* abundance over the 10 weeks of sampling with both peaking at the same time, similarly to what was observed during the 2009 monitoring in Rochambeau [23]. The best example is given by the sea urchins that were sampled over nine weeks in 2010, from week 28 to week 36. The toxins levels rose in the echinoderms WF from 17.8 to 107.6 µg PLTX eq.·kg^−1^ between week 28 and week 31 and then decreased to finally reach a concentration below the limit of quantification (LOQ) of the haemolytic test in week 36. Not all organisms could be collected on a weekly basis due to their availability *in situ*; therefore, it is not possible to generalise across all species and determine if the pattern of contamination always follows that of epiphytic cell abundances. Amzil *et al.* [22] also reported synchronicity in the contamination levels of marine organisms (mussels, sea urchins) and the presence of *O*. cf. *ovata* on macroalgae, with both reaching a peak in late July and mid-September 2009 in Morgiret Bay (Frioul islands, off the French Mediterranean coast). 

*Chelon labrosus* (thicklip grey mullet) was the only species with a maximum WF toxin concentration comprised between 20 and 30 µg PLTX eq.·kg^−1^: 27.1 µg PLTX eq.·kg^−1^ in week 29. Three species had a maximum WF toxin concentration comprised between 10 and 20 µg PLTX eq.·kg^−1^: *Eriphia verrucosa* (13.0 µg PLTX eq.·kg^−1^; week 30), *Pachygrapsus marmoratus* (16.2 µg PLTX eq.·kg^−1^; week 30) and *Octopus vulgaris* (18.3 µg PLTX eq.·kg^−1^; week 30). Eight species had a maximum WF toxin concentration comprised between the haemolytic test LOQ (1.2 µg PLTX eq.·kg^−1^) and 10 µg PLTX eq.·kg^−1^ and 13 species had WF toxin concentrations below the LOQ.

Among the 31 marine organisms sampled as part of the study, the encrusting sponges (*Antho inconstans* and *Crambe crambe*) were those with the highest WF toxin levels (90.5 and 130.5 µg PLTX eq.·kg^−1^, respectively). These high toxin levels can be attributed to two non-exclusive mechanisms: (1) the feeding behaviour of the sponges, which filter the seawater through their pores and thereby accumulate the planktonic cells in suspension in the water column and (2) epiphytic *O*. cf. *ovata* cells colonise the surface of the sponges. Despite the high toxin levels observed in these organisms—well above the EFSA threshold of 30 µg PLTX + OSTD·kg^−1^ [21]—they are not a matter of concern for public health since they are not edible.

Among the edible marine organisms, sea urchins were those with the highest WF toxin levels; the concentration in late July was 3.5 times greater than the EFSA recommendation. In 2009, sea urchins showed even higher contamination in Rochambeau with a reported WF concentration of 179.6 µg PLTX eq.·kg^−1^. It was also the most contaminated species in the other sites monitored in 2009 [23]. In the study conducted at Morgiret Bay in the same year [22], sea urchins also accumulated higher toxin levels than mussels (up to 361 µg PLTX eq.·kg^−1^ in the WF), with a marked difference by a factor or 3.3 at the end of July. Again, in summer 2011, sea urchins *Paracentrotus lividus* accumulated more PLTX-group toxins than the sampled fish species (*Sarpa salpa*, *Stramonita haemastoma*, *Mullus surmuletus*) [4], making sea urchins good sentinel species for the presence of PLTXs in marine organisms.

The Salema porgy (*Sarpa salpa*) was the second most contaminated species in our study, as reported in Rochambeau in 2011 with even higher WF concentrations (up to 152 µg PLTX eq.·kg^−1^) [4], probably in relation with the higher abundances of benthic and planktonic *Ostreopsis* cf. *ovata*. However, in 2009 the *S. salpa* had much lower WF toxin levels than other sampled marine organisms [23].

Among the 31 species sampled as part of the present study, eight had been collected the year before [23]. Other than sea urchins *P. lividus* and the *S. salpa* (see above), the spinous spider crab (*Maja squinado*), and the warty crab (*Eriphia verrucosa*) showed maximum WF toxin levels that were higher by a factor of 25.6 and 2.6, respectively, in 2010 compared with 2009. In contrast, the maximum PLTX concentration in *Patella* limpets was almost twice as high as in 2009. For the remaining three species, the octopus (*Octopus vulgaris*), the scorpion fish (*Scorpaena porcus*) and the Mediterranean moray (*Muraena helena*), the maximum WF toxin levels were similar in both studies. Regarding the fish species *Mullus surmuletus*, collected in 2010 (present study) and in 2011 [4], the samples contained no detectable amounts of toxins in the WF.

Over the 31 species sampled as part of the study, three were herbivorous, 20 carnivorous and eight omnivorous (Table 1 and Table 2). In the 10 species with the highest toxins levels, four were omnivorous (*C. crambe*, *A. inconstans*, *C. labrosus*, *P. marmoratus*), four were carnivorous (*Maja squinado*, *Hexaplex trunculus*, *O. vulgaris*, *E. verrucosa*) and two were herbivorous (*P. lividus*, juvenile *S. salpa*). The most contaminated species were the two encrusting sponges, both omnivorous filter feeders, followed in decreasing order by the two herbivorous species mentioned above (eating macroalgae or sea grasses supporting benthic *Ostreopsis*) and the carnivorous *M. squinado* and *H. trunculus*.

**Table 1:** Information regarding the fish species sampled in Rochambeau.

Group	Common Name	Species	Authorities	Tissue Analysed ^1^	Diet ^2^	Major Food Source
**Fishes**	Combtooth blenny	*Lipophrys pholis*	Linnaeus, 1758	DT, RT	O	Crustaceans, other invertebrates and plants
	Common two-banded seabream	*Diplodus vulgaris*	Geoffroy Saint-Hilaire, 1817	WF	C	Benthic invertebrates, crustaceans, worms and molluscs
	Corkwing wrasse	*Symphodus melops*	Linnaeus, 1758	WF	C	Shellfish and crustaceans
	East Atlantic peacock wrasse	*Symphodus tinca*	Linnaeus, 1758	DT, RT	C	Sea urchins, brittle stars, bivalves, shrimps and crabs
	Five-spotted wrasse	*Symphodus roissali*	Risso, 1810	WF	C	Small invertebrates like crustaceans, molluscs and sea urchins
	Gobbi	*Gobiidae*		WF	C	Most Gobiidae are carnivorous, but some feed on phytoplankton
	Lizard fish	*Saurida undosquamis*	Richardson, 1848	DT, RT	C	Fish, crustaceans, and other invertebrates
	Mediterranean moray	*Muraena helena*	Linnaeus, 1758	DT, RT	C	Fish, crayfish and cephalopods, but also dead animals
	Mediterranean rainbow wrasse	*Coris julis*	Linnaeus, 1758	WF	C	Amphipods, isopods, sea urchins, polychaetes, shrimp, and small gastropods
	Ornate wrasse	*Thalassoma pavo*	Linnaeus, 1758	WF	C	Small invertebrates including crustaceans, molluscs and worms
	Oscellated wrasse	*Symphodus ocellatus*	Linnaeus, 1758	WF	C	Benthic invertebrates including crustaceans, worms, molluscs and other small prey
	Painted comber	*Serranus scriba*	Linnaeus, 1758	WF	C	Crustaceans, molluscs and fish
	Pointed-snout wrasse	*Symphodus rostratus*	Bloch, 1791	WF	C	Small prey, mainly crustaceans
	Red mullet	*Mullus surmuletus*	Linnaeus, 1758	WF	C	Worms and crustaceans found while excavating soft substrates
	Saddled seabream	*Oblada melanura*	Linnaeus, 1758	DT, RT	O	Algae, zooplankton, small animals (crustaceans, larvae) and fish and invertebrate eggs. The proportion of planktonic prey decreases with growth in favour of benthic prey
	Salema porgy (seabream < 10 cm)	*Sarpa salpa*	Linnaeus, 1758	DT, RT	H	Feeding behaviour depending on age. Adults feed on macroalgae and *Posidonia* leaves
	Salema porgy (seabream > 20 cm)	*Sarpa salpa*	Linnaeus, 1758	DT, RT	H	
	Scorpion fish	*Scorpaena porcus*	Linnaeus, 1758	DT, RT	C	Crabs, shrimps and fish
	Thicklip grey mulet	*Chelon labrosus*	Risso, 1827	DT, RT	O	Plants, benthic microorganisms, small invertebrates and fingerlings
	White seabream	*Diplodus sargus*	Linnaeus, 1758	DT, RT	C	Crustaceans, molluscs and echinoderms

^1^ DT = digestive tube; RT = remaining tissue; WF = whole flesh. For organisms analysed by tissue component (DT, roe, RT), the toxin concentration in the WF was estimated using the toxin concentration and the weight of the different tissue components; ^2^ H = herbivorous; O = omnivorous; C = carnivorous.

**Table 2:** Information regarding marine organisms, other than fish, sampled in Rochambeau.

Group	Common Name	Species	Authorities	Tissue Analysed ^1^	Diet ^2^	Major Food Source
**Crustaceans**	Marbled crab	*Pachygrapsus marmoratus*	Fabricius, 1787	WF	O	Algae and animals, particularly mussels, limpets and members of its own species
	Spinous spider crab	*Maja squinado*	Herbst, 1788	WF	C	Molluscs and small crustaceans
	Warty/yellow crab	*Eriphia verrucosa*	Forskâl, 1775	WF	C	Opportunist, feeds on dead or live prey
	Xantho crab	*Xantho poressa*	Olivi, 1792	WF	C	Opportunist, feeds on dead or live prey
**Bivalve molluscs**	Noah’s ark	*Arca noae*	Linnaeus, 1758	WF	O	Plankton and fine organic particles filtered from the water column (via gills)
**Gastropods**	Banded dye-murex	*Hexaplex trunculus*	Linnaeus, 1758	WF	C	Feeds on various organisms (bivalves, gastropods, hermit crabs, barnacles, tunicates, worms, *etc.*) and also scavenges (mainly dead fish)
	Patella	*Patella* spp.	Linnaeus, 1758	WF	H	Microalgae and cyanobacteria found on the substrate they live on
**Echinoderms**	Sea urchin	*Paracentotus lividus*	Lamarck, 1816	DT, roe	H	Macroalgae
**Cephalopods**	Octopus	*Octopus vulgaris*	Cuvier, 1797	DT, RT	C	Crustaceans and molluscs
**Sponges**	Elephant’s hide sponge	*Pachymatisma johnstonia*	Bowerbank in Johnston, 1842	WF	O	Bacteria, organic debris and unicellular algae
	Orange-red encrusting sponge	*Crambe crambe*	Schmidt, 1862	WF	O	
	Red encrusting sponge	*Antho inconstans*	Topsent, 1925	WF	O	

^1^ DT = digestive tube; RT = remaining tissue; WF = whole flesh. For organisms analysed by tissue component (DT, roe, RT), the toxin concentration in the WF was estimated using the toxin concentration and the weight of the tissue components; ^2^ H = herbivorous; O = omnivorous; C = carnivorous.

Based on the data reported in the present study, there is a relationship between diet and contamination level in marine organisms. The four most contaminated species were omnivorous or herbivorous (*C. crambe*, *A. inconstans*, *P. lividus*, juvenile *S. salpa*) and their feeding behaviour puts them in direct and close contact with planktonic and epiphytic *Ostreopsis* cells (sponges as filter feeding organisms, *P. lividus* and *S. salpa* as the two main macro-herbivores in the Mediterranean Sea). Brissard *et al*. [4] also reported that herbivores (*P. lividus* and *S. salpa*) grazing on macroalgae covered with *O*. cf. *ovata* accumulate higher toxin levels than carnivorous species sampled as part of the study, further confirming suggestions that diet and contamination levels are correlated in species sampled in Rochambeau in 2009 [23]. However, some carnivorous organisms such as *M. squinado* and *H. trunculus* accumulated significant toxin levels (above the EFSA-recommended threshold), higher than other omnivorous and herbivorous species, most likely after feeding on contaminated prey. The most contaminated carnivorous organisms are known to feed on filter feeding molluscs and on crabs. Carnivores, such as *M. helena* and *S. porcus*, known to feed mainly on fish contain much less PLTX-group toxins.

During the 2009 sampling campaign in Rochambeau, juvenile *S. salpa* were spotted eating *Ostreopsis* cell aggregates floating in the water column, while they generally graze on macroalgae, like the adults [23]. In 2010 there was no overlap between the sampling periods of juvenile (<10 cm) and adult (>20 cm) *S. salpa*; the adult specimens were sampled from week 27 to week 30 with WF toxins levels below the LOQ, while the juveniles were harvested in the following weeks (31 to 36) and showed higher concentrations (ranging from the LOQ to 71.8 µg PLTX eq.·kg^−1^). Although the contamination levels cannot be compared *per se* due to different sampling periods, the *Ostreopsis* abundances were similar in weeks 30 and 31 (*ca*. 233,000 cells·g^−1^ FW) but the WF toxin content of adult *S. salpa* (week 30: 0.9 and 1.9 µg·kg^−1^ using the lower (LB) and upper bound (UB) approach, respectively) was much lower than that observed in the juveniles (week 31: 71.8 and 72.3 µg·kg^−1^, respectively). This difference can also be attributed partly or completely to the fact that the juveniles sampled in week 31 were exposed to *Ostreopsis* cells for an extra week compared to the adults. Therefore, we cannot conclude as to whether the feeding behaviour influences the toxin level in juvenile and adult *S. salpa*. Juvenile *S. salpa* were also found to accumulate high toxin concentrations in Rochambeau in 2011, with levels two times higher than those reported here; however no comparison was made with adult specimens in that study [4]. Given that juvenile *S. salpa* can feed on cell aggregates in the water column and graze on macroalgae covered with epiphytic *Ostreopsis* cells like the adults, this fish species is potentially exposed to toxigenic phytoplankton in all its life stages, making it a good sentinel species to monitor PLTX-group toxins in fish, with a preference for juveniles over adults. Juveniles eat often at low depth while adults can move over longer distances (and deeper) and also graze on seagrasses (*Posidonia oceanica*).

**Figure 2:**
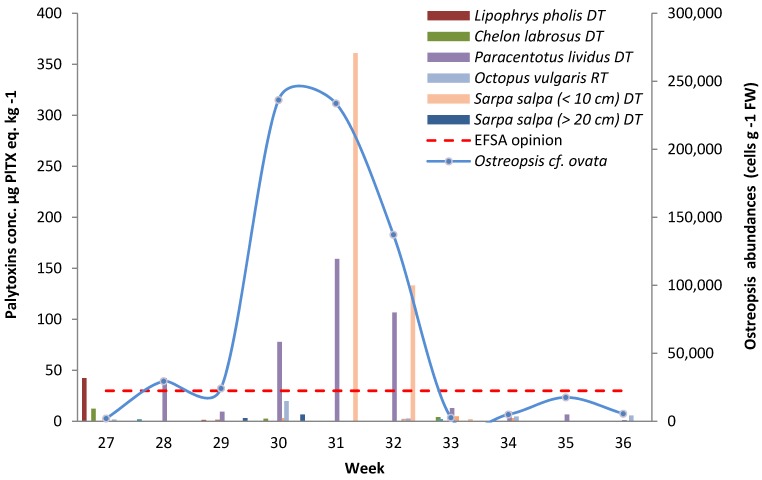
Palytoxin concentration determined by the haemolytic test in the different tissue components of the marine organisms harvested in Rochambeau from week 27 to week 36. The dashed line represents the EFSA threshold of 30 µg PLTX + OST-D·kg^−1^. The blue curve represents the abundance of *Ostreopsis* epiphytic cells over the same period.

The analysis of some of the samples collected was separated into different tissue components to study the body distribution of palytoxins. The data presented in Figure 2 show that toxins are sequestered in the digestive tube (DT) of the analysed species, but not in the roe of *P. lividus* nor in the remaining tissues (RT < LOQ), except for O. vulgaris which preferentially accumulated palytoxins in the RT (week 30: 19.9 µg PLTX eq.·kg^−1^) and much less in the DT (week 30: 3.3 µg PLTX eq.·kg^−1^).

The highest toxin level was found in the DT of juvenile *S. salpa* with 361.0 µg PLTX eq.·kg^−1^ in week 31. A week later, the concentration had decreased by a factor of three but was still more than four times higher than the EFSA threshold of 30 µg PLTX+OST-D·kg^−1^. Adult *S. salpa* had much lower toxin levels in the DT, comprised between the LOQ and 6.8 µg PLTX eq.·kg^−1^ (week 30).

*P. lividus* also had important toxin levels in the DT, but not in the roe (<LOQ). On four occasions, the concentrations were greater than 30 µg PLTX eq.·kg^−1^ (week 28: 42.5; week 30: 78.0; week 31: 159.2; week 32: 106.7 µg PLTX eq.·kg^−1^).

In week 27, the fish *C. labrosus* and *Lipophrys pholis* had toxin levels of 12.5 and 42.4 µg PLTX eq.·kg^−1^ in their DTs, respectively, but their RTs were not contaminated (<LOQ).

The analysis of the different tissue components (DT, RT/roe) of marine organisms revealed a general pattern: DT was the most and only contaminated part for the vast majority of the species, whatever the group they belonged to (echinoderms, fish), confirming previous observations at the same site and for the same species [23], as well as in 2011 for *P. lividus* and the *S. salpa* [4]. In 2010, the most contaminated DT was that of the juvenile *S. salpa*, whereas, in the year before, the toxin level in the DT of this species was 14 times lower [23]. Another common feature between the 2009 and 2010 studies was the higher toxin levels in the RT than in the DT in the common octopus. The hypothesis of *Ostreopsis* cells colonizing the octopus RT, especially the tentacles that come into contact with epiphytic cells, is unlikely as they were thoroughly rinsed in clear water before sample preparation and analysis. Like the other contaminated carnivorous species in this study, the common octopus mainly feeds on crustaceans and molluscs, and not on fish. Cephalopods may accumulate different marine biotoxins in their tissues, including domoic acid, saxitoxins and PLTX-group toxins, with levels of p-PLTX and OVTX-a that can reach 115 and 971 µg·kg^−1^, respectively [27].

### 2.2. Mean Toxin Concentrations in Marine Organisms

The mean palytoxin levels detected in the sampled species are given in Table 3 for the marine organisms consumed as whole flesh and in Table 4 for those consumed as RT, *i.e.*, eviscerated. They were determined according to the LB and UB approach to deal with the left-censored (LC) values. LC values accounted for 46% (57 out of 123) of the data for the species consumed as WF and 83% (35 out of 43) of the data consumed as RT or roe.

#### 2.2.1. Marine Organisms Consumed as Whole Flesh

*Fish*: the LC values accounted for 61% (46 out of 75) of the data for this group (Table 3). The mean palytoxin concentrations in the different fish species ranged from 0.0 to 19.4 µg PLTX eq.·kg^−1^ and from 1.2 to 20.1 µg PLTX eq.·kg^−1^ for the LB and the UB approach, respectively. The highest mean toxin concentration was reported for juvenile *S. salpa*. The corresponding group means were 2.0 and 3.0 µg PLTX eq.·kg^−1^, respectively and 3% of the reported results (two samples of juvenile *S. salpa* collected in weeks 31 and 32) were above the threshold of 30.0 µg PLTX eq.·kg^−1^.

*Crustaceans*: the LC values accounted for 38% (6 out of 16) of the data for this group. The mean palytoxin concentrations in the crustacean species ranged from 3.6 to 22.6 µg PLTX eq.·kg^−1^ and from 3.6 to 23.0 µg PLTX eq.·kg^−1^ for the LB and the UB approach, respectively. The highest toxin concentration was reported for the *M. squinado* (51.3 PLTX eq.·kg^−1^). The corresponding total group means were 8.7 and 9.0 µg PLTX eq.·kg^−1^, respectively and 6% of the reported results (one *M. squinado* sample collected in week 31) were above the threshold of 30.0 µg PLTX eq.·kg^−1^.

*Bivalve molluscs*: the LC values accounted for 0% (0 out of 2) of the group data, with *Arca noae* as the only group member. The mean palytoxin concentration was 3.8 µg PLTX eq.·kg^−1^ for both the LB and the UB approach. None of the reported results was above the threshold of 30.0 µg PLTX eq.·kg^−1^.

*Gastropods*: the LC values accounted for 31% (5 out of 16) of the data for this group. The mean palytoxin concentrations in the different gastropod species ranged from 2.4 to 7.3 µg PLTX eq.·kg^−1^ and from 2.8 to 7.7 µg PLTX eq.·kg^−1^ for the LB and the UB approach, respectively. The highest mean toxin concentration was observed in *H. trunculus*. The corresponding total group means were 4.9 and 5.3 µg PLTX eq.·kg^−1^, respectively and less than 6% of the reported results (one *H. trunculus* sample collected in week 32) were above the threshold of 30.0 µg PLTX eq.·kg^−1^. 

*Echinoderms*: the LC values accounted for 0% (0 out of 9) of the group data, with *P. lividus* as the only group member. The mean palytoxin concentration was 30.3 and 30.7 µg PLTX eq.·kg^−1^ for the LB and the UB approach, respectively; this is the only case where the mean toxin concentration was above 30.0 µg PLTX eq.·kg^−1^. Of the individually reported results, 33% (three sea urchins samples collected in week 30, 31 and 32) were *ca.* 60% to 230% above the threshold of 30.0 µg PLTX eq.·kg^−1^.

**Table 3:** Mean palytoxin (PLTX)-group toxin concentrations (µg·kg^−1^) determined by the haemolytic test in the whole flesh (WF) of fishery products harvested in Rochambeau from week 27 to week 36.

Species	Common Name	Tissue Analysed	*N*	%LC	Mean		Min	Max	>30 µg·kg^−1^
					LB	UB	LB/UB	LB/UB	%
**Fish**	*Lipophrys pholis*	WF	1	0%	8.3	9.3	8.3/9.3	8.3/9.3	0%
	*Diplodus vulgaris*	WF	4	0%	2.9	3.2	1.3/1.3	4.2/4.8	0%
	*Symphodus melops*	WF	1	100%	0.0	1.2	0.0/1.2	0.0/1.2	0%
	*Symphodus tinca*	WF	6	83%	0.2	1.2	0.0/1.2	0.0/1.2	0%
	*Symphodus roissali*	WF	6	83%	0.2	1.2	0.0/1.2	0.0/1.2	0%
	*Gobiidae*	WF	1	0%	1.1	1.1	1.1/1.1	1.1/1.1	0%
	*Saurida undosquamis*	WF	1	0%	0.2	1.2	0.2/1.2	0.2/1.2	0%
	*Murena helena*	WF	4	100%	0.0	1.2	0.0/1.2	0.0/1.2	0%
	*Coris julis*	WF	7	43%	0.9	1.6	0.0/1.2	2.8/2.8	0%
	*Thalassoma pavo*	WF	2	100%	0.0	1.2	0.0/1.2	0.0/1.2	0%
	*Symphodus ocellatus*	WF	1	100%	0.0	1.2	0.0/1.2	0.0/1.2	0%
	*Serranus scriba*	WF	3	100%	0.0	1.2	0.0/1.2	0.0/1.2	0%
	*Symphodus rostratus*	WF	2	100%	0.0	1.2	0.0/1.2	0.0/1.2	0%
	*Mullus surmuletus*	WF	3	67%	0.3	1.3	0.0/1.2	0.9/1.5	0%
	*Oblada melanura*	WF	3	100%	0.0	1.2	0.0/1.2	0.0/1.2	0%
	*Sarpa salpa* (juveniles < 10 cm)	WF	6	33%	19.4	20.1	0.0/1.2	71.8/72.3	33%
	*Sarpa salpa* (adults > 20 cm)	WF	3	33%	0.6	1.6	0.1/1.2	0.9/1.9	0%
	*Scorpaena porcus*	WF	7	71%	0.6	1.5	0.0/1.2	2.3/2.3	0%
	*Chelon labrosus*	WF	7	14%	5.5	7.2	0.0/1.2	27.1/27.1	0%
	*Diplodus sargus*	WF	7	86%	0.5	1.6	0.0/1.2	3.7/3.7	0%
	***Total group mean***	***WF***	***75***	***61%***	***2.0***	***3.0***	***0.0/1.2***	***71.8/72.3***	***3%***
**Crustaceans**	*Pachygrapsus marmoratus*	WF	7	43%	4.5	5.0	0.0/1.2	16.2/16.2	0%
	*Maja squinado*	WF	3	33%	22.6	23.0	0.0/1.2	51.3/51.3	33%
	*Eriphia verrucosa*	WF	5	40%	3.9	4.4	0.0/1.2	13.0/13.0	0%
	*Xantho poressa*	WF	1	0%	3.6	3.6	3.6/3.6	3.6/3.6	0%
	***Total group mean***	***WF***	***16***	***38%***	***8.7***	***9.0***	***0.0/1.2***	***51.3/51.3***	***6%***
**Bivalve molluscs**	*Arka noae*	WF	2	0%	3.8	3.8	2.3/2.3	5.2/5.2	0%
**Gastropods**	*Hexaplex trunculus*	WF	8	25%	7.3	7.7	0.0/1.2	40.4/40.4	13%
	*Patella* spp.	WF	8	38%	2.4	2.8	0.0/1.2	6.6/6.6	0%
	***Total group mean***	***WF***	***16***	***31%***	***4.9***	***5.3***	***0.0/1.2***	***40.4/40.4***	***6%***
**Echinoderms**	*Paracentrotus lividus*	WF	9	0%	30.3	30.7	0.8/1.3	107.6/108.0	33%
**Cephalopods**	*Octopus vulgaris*	WF	5	0%	6.1	6.3	0.5/1.6	18.3/18.3	0%
***Overall group mean***		***WF***	***123***	***46%***	***4.3***	***5.1***	***0.0/1.2***	***107.6/108.0***	***<1%***

*N* = number of samples collected during the 10-week sampling period; LC = left censored data; LB = lower bound; UB = upper bound; WF = whole flesh. For organisms analysed by tissue component (DT, roe, RT), the toxin concentration in the WF was estimated using the toxin concentration and the weight of the different tissues.

**Table 4:** Mean palytoxin concentrations (µg·kg^−1^) determined with the haemolytic test in the remaining tissues (RT) and roe of marine organisms harvested in Rochambeau from week 27 to week 36.

Species	Common Name	Tissue Analysed	*N*	%LC	Mean		Min	Max	>30 µg·kg^−1^
					LB	UB	LB/UB	LB/UB	%
**Fish**	*Lipophrys pholis*	RT	1	100%	0.0	1.2	0.0/1.2	0.0/1.2	0%
	*Symphodus tinca*	RT	2	50%	1.0	1.6	0.0/1.2	2.0/2.0	0%
	*Saurida undosquamis*	RT	1	100%	0.0	1.2	0.0/1.2	0.0/1.2	0%
	*Muraena helena*	RT	4	100%	0.0	1.2	0.0/1.2	0.0/1.2	0%
	*Oblada melanura*	RT	2	100%	0.0	1.2	0.0/1.2	0.0/1.2	0%
	*Sarpa salpa* (juvenile < 10 cm)	RT	4	100%	0.0	1.2	0.0/1.2	0.0/1.2	0%
	*Sarpa salpa* (adult > 20 cm)	RT	3	100%	0.0	1.2	0.0/1.2	0.0/1.2	0%
	*Scoprpaena porcus*	RT	6	83%	0.2	1.2	0.0/1.2	1.4/1.4	0%
	*Chelon labrosus*	RT	3	67%	0.7	1.5	0.0/1.2	2.2/2.2	0%
	*Diplodus sargus*	RT	2	100%	0.0	1.2	0.0/1.2	0.0/1.2	0%
	***Total group mean***	***RT***	***28***	***89%***	***0.2***	***1.3***	***0.0/1.2***	***2.2/2.2***	***0%***
**Echinoderms**	*Paracentrotus lividus*	Roe	9	100%	0.0	1.2	0.0/1.2	0.0/1.2	0%
**Cephalopods**	*Octopus vulgaris*	RT	5	20%	6.5	6.7	0.0/1.2	19.9/19.9	0%
***Overall group mean***		***RT/Roe***	***42***	***83%***	***0.7***	***1.7***	***0.0/1.2***	***19.9/19.9***	***0%***

*N* = number of samples collected during the 10-week sampling period; LC = left censored data; LB = lower bound; UB = upper bound; RT = remaining tissue.

*Cephalopods*: the LC values accounted for 0% (0 out of 5) of the group data, with *O. vulgaris* as the only group member. The mean palytoxin concentration was 6.1 and 6.3 µg PLTX eq.·kg^−1^ for the LB and the UB approach, respectively. None of the reported results were above the threshold of 30.0 µg PLTX eq.·kg^−1^.

Given that PLTX-group toxins accumulate in the DT, but not the RT or roe of the tested organisms (*L. pholis*, *Saurida undosquamis*, *C. labrosus*, *Oblada melanura*, *S. porcus*, *Diplodus sargus*, *S. salpa*, *P. lividus*), except in the case of *O. vulgaris*, eating marine organisms as WF theoretically exposes consumers to higher toxin levels. However, the overall group mean PLTX concentration was roughly six to seven times lower than the EFSA-recommended threshold. None of the different groups of marine organisms had the same number of species (from one for bivalve molluscs, echinoderms and cephalopods to 19 for fish), causing the statistical power to vary. The EFSA opinion [21] is based on the results reported for p-PLTX and OVTX-a in shellfish meat; the mean concentration levels reported whatever the approach used (LB or UB) are higher than those observed here, with mean toxin values for all species exceeding 30 µg·kg^−1^ by a factor of 2.8 to 3.8. The LC values are of the order of those observed in the present study, ranging from 0.0% to 58.2% of the reported values for the PLTX-group toxins depending on the detection method used. The EFSA data were derived from mussels and sea urchins, two species described as capable of accumulating high levels of PLTX-group toxins [4,22,23,28,29], and this is probably why the mean values reported in the EFSA opinion are higher. Therefore, the nature and the quality of the data can influence conclusions with respect to risk assessment. EFSA based its opinions on the available data provided by the European Union (EU) Member States, and at the time of the call for data, only three Member States reported data, and on only a very limited number of species. More data on different marine organisms (fish, bivalves, crustaceans, echinoderms, gastropods *etc.*) is required to refine the EFSA opinion.

#### 2.2.2. Marine Organisms Consumed as Remaining Tissues

*Fish*: the LC values accounted for 89% (25 out of 28) of the data for this group (Table 4). The mean palytoxin concentrations in the different fish species ranged from 0.0 to 1.0 µg PLTX eq.·kg^−1^ and from 1.2 to 1.6 µg PLTX eq.·kg^−1^ for the LB and the UB approach, respectively. The highest mean toxin concentration was observed in *Symphodus tinca*. The corresponding total group means were 0.2 and 1.3 µg PLTX eq.·kg^−1^, respectively.

*Echinoderms*: the LC values accounted for 100% (9 out of 9) of the data for this group, with *P. lividus* as the only group member. The mean palytoxin concentration in roe was 0.0 and 1.2 µg PLTX eq.·kg^−1^ for the LB and the UB approach, respectively.

*Cephalopods*: the LC values accounted for 20% (0 out of 5) of the data for this group, with *O. vulgaris* as the only group member. The mean palytoxin concentration was 6.5 and 6.7 µg PLTX eq.·kg^−1^ for the LB and the UB approach, respectively. 

The mean PLTX concentrations in the marine organisms consumed as RT reflect the fact that p-PLTX and its analogues generally accumulate in the DT. This distribution pattern has been described and confirmed elsewhere [4,23] and indicates that the evisceration process helps decrease the risk of exposure to potentially dangerous levels of PLTX-group toxins in marine organisms. In the case of sea urchins, the roe is the preferred part but some people eat the TD and roe, altogether. Furthermore, *Ostreopsis* bloom occurs in summer, while harvesting edible sea urchin is prohibited in many NW Mediterranean countries; however poaching is possible. Therefore the risk associated to PLTX-group toxins in edible sea urchins has to be taken into account.

### 2.3. Comparison of the Toxin Levels Determined by Haemolytic Test and LC-MS/MS

LC-MS/MS analysis was used for confirmatory purposes on all samples screened by the haemolytic test with a toxin concentration above the LC-MS/MS LOQ of 24.5 µg PLTX eq.·kg^−1^.

**Figure 3:**
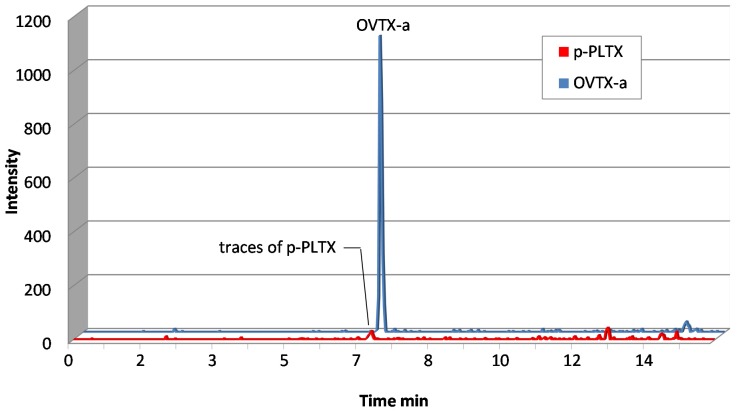
Chromatogram of a sample of juvenile *Sarpa salpa* DT harvested in week 31 and analysed by LC-MS/MS, showing traces of p-PLTX (<LOQ) and a high level of OVTX-a (405 µg PLTX eq.·kg^−1^).

**Figure 4:**
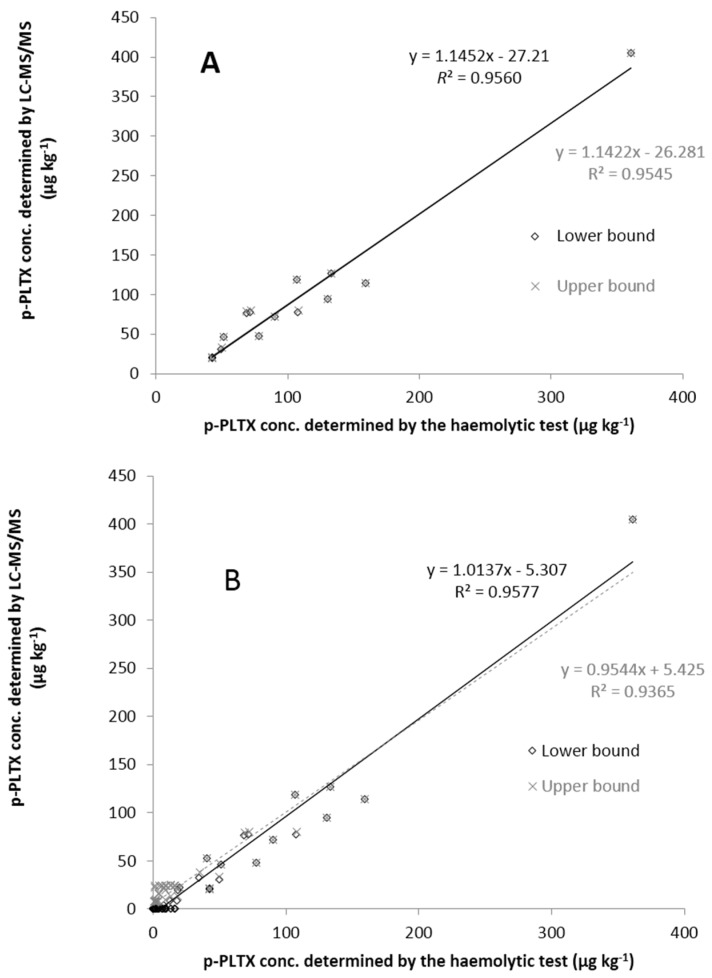
Relationship between the palytoxin levels determined by the haemolytic test and by LC-MS/MS. (**A**) with the LC-MS/MS LOQ (24.5 µg p-PLTX eq.·kg^−1^) as the data cut-off point; (**B**) without the cut-off point (all data included even those below the LC-MS/MS LOD (7.4 µg p-PLTX eq.·kg^−1^) and LOQ, for which the LB and UB approach was used).

The toxin profile of the samples analysed by LC-MS/MS was exclusively composed of OVTX-a (Figure 3); none of the other toxins (p-PLTX, OST-D, OVTX-a to -d) were either detected or quantified. As shown in Figure 4, there is a good linear association between the results obtained with both methods, with coefficients of determination (*R*^2^) above 0.93. The strength of the relationship between the two methods was higher when the datasets had a cut-off point equal to the LC-MS/MS LOQ (*R*^2^ of 0.9560 and 0.9545 for the LB and UB approach, respectively). 

As reported in previous studies [4,23], there is good agreement between both techniques, LC-MS/MS and the haemolytic test. However, there are situations in which the agreement between both sets of results is not so strong. Brissard *et al.* [4] reported matrix effects when analysing samples of *S. salpa* by LC-MS/MS but not with the haemolytic test. The difference in sensitivity of both techniques also contributes, to a certain extent, to the discrepancy of the results especially for the UB approach. In contrast to the LB approach, in which the limit of detection (LOD) and LOQ values are assigned a value of zero, the actual values of LOD and LOQ are used in the UB approach. Therefore, the difference in the results will be increased when analysing data obtained from techniques with significantly different LODs. This explains why the linear association between LC-MS/MS and the haemolytic test was slightly higher for the results obtained using the LB approach.

## 3. Experimental Section

### 3.1. Sampling Location

The sampling site, called Rochambeau (43°41′34.83″ N and 7°18′31.66″ E), in the Bay of Villefranche was chosen based on its history of occurrence of *Ostreopsis* cf. *ovata* and the level of PLTX-group toxins in marine organisms recorded in a previous study [23].

### 3.2. Sampling Periods

The marine organisms targeted in the study as well as *Ostreopsis* cf. *ovata* samples were collected weekly between 5 July 2010 (week 27) and 12 September 2010 (week 36). 

*Ostreopsis* sampling strategy and counting methods are described elsewhere [23]. Epiphytic *Ostreopsis* was sampled in four sub-sites spaced by 10 m, mainly using the macroalgae *Halopteris scoparia* (Linnaeus) Sauvageau, or (but very rarely) *Corallina* spp. The *Ostreopsis* abundances are expressed as the average of the cell counts in the four different sampling points in Rochambeau.

### 3.3. Sampling of Marine Organisms

A wide variety of marine organisms were present and sampled, including molluscs (gastropods and cephalopods), echinoderms, fish, crustaceans and sponges (Table 1 and Table 2). The status of scientific names and authorities were checked in WoRMS (World Register of Marine Species; www.marinespecies.org). All species, except sponges, are edible on their own or as an ingredient, e.g., in a fish soup.

### 3.4. Sampling Techniques and Sample Preparation

All species were collected by snorkelling or fishing from the shore, from the surface to 3 m depth maximum. Depending on the species, a variable number of specimens were sampled to obtain a sufficient amount of tissue for toxin analysis, but in moderation according to the existing population.

Ten fish species and all molluscs and crustaceans were analysed in terms of WF, *i.e.*, after removing the head, the fish bones, the mollusc shell or the crustacean exoskeleton. For all other marine organisms (cephalopods, echinoderms and the remaining nine fish species), the DT was separated from the RT or roe in the case of the sea urchins *P. lividus*, and the tissue components were analysed separately. The WF toxin concentration, composed of the DT and the roe for *P. lividus* and DT and RT for the other species (except molluscs and crustaceans, analysed as whole organisms and not as different parts), was then calculated as the sum of the toxin concentration of the different tissues weighted by the weight (in gram) of the corresponding tissues.

All the samples were stored at −20 °C until analysis using the haemolytic test and LC-MS/MS.

### 3.5. Determination of the Toxin Content of the Marine Organisms

#### 3.5.1. Reagents Used

All reagents were of analytical grade unless otherwise specified.

PLTX was purchased from Wako Chemical GmbH (Neuss, Germany) as a 100 µg lyophilised powder. The toxin quantity was determined gravimetrically. This standard was 90% pure as estimated chromatographically.

De-ionised water (18.2 mΩ) was obtained using a Milli-Q^®^ purification system (Millipore, Molsheim, France).

Methanol (MeOH) and acetonitrile (MeCN) were of HPLC grade and were purchased from Fisher Scientific (Illkirch, France).

Mouse blood containing lithium heparinate as an anti-blotting agent was purchased from Charles River Laboratories (L’Arbresle, France).

#### 3.5.2. Haemolytic Test

The haemolytic test is based on the capacity of PLTX and analogues to convert the Na^+^/K^+^-ATPase pump into a non-specific channel, leading to ion imbalance and delayed haemolysis of mammalian erythrocytes.

A series of 11 calibration points was prepared using the PLTX standard, with concentrations ranging from 0.0 (blank) to 5.0 ng/mL (1.9 pM). Similarly, the sample extracts had to be diluted several folds to enable the quantification of the toxin content. The dilution factors applied were comprised between 1/2 and 1/256. All dilutions were made in 30% aqueous MeOH.

A 50 mL aliquot of the diluted extract or calibration point was added to 950 mL of phosphate buffer saline (PBS) containing 0.5% (v/v) of mouse erythrocytes obtained from Charles River Laboratories (L’Arbresle, France). The number of erythrocytes in the PBS buffer containing 0.5% of mouse erythrocytes was checked as a quality control; the average count over 131 determinations was 5.7 × 10^7^ cells (relative standard deviation = 9.7%). The solution was incubated at 37 °C for 4 h and centrifuged at 1000× *g* for 10 min. A 200 mL aliquot of each extract or calibration point was put in triplicate in a 96-well plate (Nunc microwell F96 PS NST, Fisher Scientific, Illkirch, France) and the optical density (OD) was read at 450 nm with a spectrophotometer (MRX 3100, Dynex Technologies, Denkendorf, Germany). The percentage of lysis is a linear function of the OD (ratio of the difference between OD and the blank OD (OD min) and the difference between the maximum OD and the blank OD). The relationship between the percentage of lysis and the concentration of palytoxin is a sigmoidal curve that is transformed in a linear curve by using the logit/log model. The percentage of lysis of an extract was determined from the OD obtained for each dilution of this extract. The toxin concentration in the extract was determined using the logit/log model and was calculated using the linear part of the curve Logit (% lysis) = a·log *X* + b, giving the following equations:

concentration *X* (in ng/mL) =10^(Logit − b)/a)^ × dilution factor

with $\mathrm{Logit}=\ln\;\frac{\text{\% }\mathrm{lysis}}{\text{1 - \% }\mathrm{lysis}}$ and $\text{\%lysis = }\frac{{\mathrm{OD}\text{ - }\mathrm{OD}}_{\min}}{{\mathrm{OD}}_{\max}\;{\text{- }\mathrm{OD}}_{\min}}{\text{ = }\mathrm{slope}}_{\mathrm{calibration}\;\mathrm{curve}}{\text{ × OD + }\mathrm{intercept}}_{\mathrm{calibration}\;\mathrm{curve}\;}$.

As the haemolytic potency was estimated using PLTX as a calibrant, the results were expressed in microgram of PLTX equivalent per kilogram of the analysed matrix (µg PLTX eq.·kg^−1^). The LOQ of the haemolytic test was determined as 1.2 µg PLTX eq.·kg^−1^.

The specificity of the haemolytic activity of PLTX was confirmed on the positive samples by adding 50 µL aliquot of the diluted extract or calibration point to 950 µL phosphate buffer saline containing 0.5% (v/v) of mouse erythrocytes and 500 µM of ouabain pre-incubated at 37 °C for 1 h. In the presence of PLTX and ouabain a shift in the response curve compared to the curve without ouabain was observed.

The experimental details regarding the extraction procedure have been published elsewhere [23].

#### 3.5.3. Tandem Mass Spectrometry (LC-MS/MS)

LC-MS/MS analyses were carried out on a Dionex Ultimate 3000 (Dionex, Villebon-sur Yvette, France) LC system coupled to an API4000 Qtrap mass spectrometer (AB Sciex, Les Ulis, France), as described by Biré *et al.* [23] with the following modifications: p-PLTX and PLTX-analogues, OVTX-a to -d and OST-D, were analysed in positive mode with an ionspray voltage of 5.5 kV. MRM experiments were carried out by selecting two transitions (precursor ion → product ion). The transitions monitored for the presence of p-PLTX and its analogues (OST-D, OVTX-a to -d) are presented in Table 5. The most intense transition was used for quantification purposes ([M + 2H − H_2_O]^2+^ → [A moiety + H − H_2_O]^+^).

**Table 5:** Liquid chromatography tandem mass spectrometry (LC-MS/MS) transitions of the PLTX-group toxins.

Toxins	[M + 2H]^2+^ → [A moiety + H − H_2_O]^+^	[M + 2H − H_2_O]^2+^ → [A moiety + H − H_2_O]^+^
p-PLTX	1340.3 → 327.3	1331.3 → 327.3
OST-D	1318.3 → 327.3	1309.3 → 327.3
OVTX-a	1324.3 → 327.3	1315.3 → 327.3
OVTX-b	1346.3 → 371.2	1337.3 → 371.2
OVTX-c	1354.3 → 371.2	1345.3 → 371.2
OVTX-d	1332.3 → 327.3	1323.3 → 327.3

The toxin concentrations in the analysed samples were determined from an external PLTX calibration curve. OVTX-a to -d and OST-D were quantified using PLTX as the calibrant, assuming an equi-molar response for all seven toxins.

The LOD and the LOQ of the LC-MS/MS method were determined as 7.4 and 24.5 µg PLTX eq.·kg^−1^, respectively.

The A moiety refers to the structure of the toxins as presented in Ciminiello *et al.* [30].

#### 3.5.4. Data Treatment

LC data are those expressed as below the LOD and below the LOQ. These data were treated by the substitution method as recommended in the Principles and Methods for the Risk Assessment of Chemicals in Food [31]. The same method is indicated in the EFSA scientific report Management of left-censored data in dietary exposure assessment of chemical substances [32] as an option in the treatment of left-censored data. Both guides suggest that the LB and UB approach should be used for chemicals likely to be present in food (e.g., naturally occurring contaminants, nutrients and mycotoxins). At the LB, results below the LOQ and LOD are assigned a value of zero; at the UB the results below the LOD are assigned the LOD value and those below the LOQ are assigned the LOQ value.

## 4. Conclusions

The analysis of a wide variety of marine organisms collected in the Bay of Villefranche in summer 2010 showed that the levels in PLTX-group toxins occasionally exceeded the EFSA-recommended threshold for several species analysed as WF. However, the mean toxin value determined for each animal group using the LB or the UB approach was below 30 µg·kg^−1^, except for sea urchins *P. lividus*. This makes the monitored echinoderms a good sentinel species to follow the presence of toxins in the environment.

The most contaminated organisms were either herbivorous or omnivorous. Some carnivorous species also contained PLTX-group toxins, but this concerned organisms that mainly feed on crustaceans and molluscs, and less on fish. The influence of diet and age on contamination in *S. salpa* requires further investigation to determine whether juveniles are indeed more contaminated than adults. 

The analysis of the different tissue components of the organisms confirmed the preferential accumulation of toxins in the DT of the tested organisms, except for *O. vulgaris*. Therefore, evisceration can be an effective procedure for managing health risks and protecting consumers from exposure to PLTX-group toxins in marine organisms. At this stage, further studies must consider eating habits and, for non-eviscerated species, depuration studies can determine the kinetics involved and possible remediation processes to decrease the toxin level in the food source. Concerning edible sea urchins, although harvesting is regulated during the summer, when *Ostreopsis* is likely to bloom, poaching and local eating habits could potentially lead to food poisonings.

Further studies on PLTX analogues are needed to assess their haemolytic activity as well as their toxicity.

## Supplementary Files

Supplementary File 1
