# Supplementary material for: Hunt for Palytoxins in a Wide Variety of Marine Organisms Harvested in 2010 on the French Mediterranean Coast
Source: Mar Drugs. 2015 Aug 21;13(8):5425–46. doi: 10.3390/md13085425 (PMC4557029; doi:10.3390/md13085425)
Supplement: Supplementary File 1 [file marinedrugs-13-05425-s001.docx]

Supplementary Materials

**Figure S1.** Abundances of the *Ostreopsis* *cf*. *ovata* planktonic cells in the water column in Rochambeau from week 27 to week 36.

© 2015 by the authors; licensee MDPI, Basel, Switzerland. This article is an open access article distributed under the terms and conditions of the Creative Commons Attribution license (http://creativecommons.org/licenses/by/4.0/).
